# 24-Hydroxycholesterol Induces Tau Proteasome-Dependent Degradation via the SIRT1/PGC1α/Nrf2 Pathway: A Potential Mechanism to Counteract Alzheimer’s Disease

**DOI:** 10.3390/antiox12030631

**Published:** 2023-03-03

**Authors:** Gabriella Testa, Serena Giannelli, Barbara Sottero, Erica Staurenghi, Giorgio Giaccone, Paola Caroppo, Paola Gamba, Gabriella Leonarduzzi

**Affiliations:** 1 Department of Clinical and Biological Sciences, University of Turin, Orbassano, 10043 Turin, Italy; 2 Division of Neurology 5 and Neuropathology, Fondazione IRCCS Istituto Neurologico Carlo Besta, 20133 Milan, Italy

**Keywords:** 24-hydroxycholesterol, sirtuin 1, Nrf2, tau, Alzheimer’s disease, proteasome

## Abstract

Considerable evidence indicates that cholesterol oxidation products, named oxysterols, play a key role in several events involved in Alzheimer’s disease (AD) pathogenesis. Although the majority of oxysterols causes neuron dysfunction and degeneration, 24-hydroxycholesterol (24-OHC) has recently been thought to be neuroprotective also. The present study aimed at supporting this concept by exploring, in SK-N-BE neuroblastoma cells, whether 24-OHC affected the neuroprotective SIRT1/PGC1α/Nrf2 axis. We demonstrated that 24-OHC, through the up-regulation of the deacetylase SIRT1, was able to increase both PGC1α and Nrf2 expression and protein levels, as well as Nrf2 nuclear translocation. By acting on this neuroprotective pathway, 24-OHC favors tau protein clearance by triggering tau ubiquitination and subsequently its degradation through the ubiquitin–proteasome system. We also observed a modulation of SIRT1, PGC1α, and Nrf2 expression and synthesis in the brain of AD patients with the progression of the disease, suggesting their potential role in neuroprotection. These findings suggest that 24-OHC contributes to tau degradation through the up-regulation of the SIRT1/PGC1α/Nrf2 axis. Overall, the evidence points out the importance of avoiding 24-OHC loss, which can occur in the AD brain, and of limiting SIRT1, PGC1α, and Nrf2 deregulation in order to prevent the neurotoxic accumulation of hyperphosphorylated tau and counteract neurodegeneration.

## 1. Introduction

Alzheimer’s disease (AD) is the most common form of progressive dementia in the elderly population, characterized by cognitive impairment, memory loss, and behavioral abnormalities [1].

Due to the difficulty of early diagnosis and to the lack of effective therapeutic methods, more incentives are needed to better elucidate the crucial mechanisms involved in AD pathogenesis that could be targets for the development of new strategies to prevent or slow down this disabling disease. In this connection, many mechanisms have been proposed to contribute to AD, including mitochondrial dysfunction, oxidative stress, neuroinflammation, and cholesterol and glucose dysmetabolism [2,3,4,5,6,7,8,9].

Of note, the dysfunction of protein degradation pathways has been recently proposed to play an important role in AD. Cellular aggregation and the accumulation of abnormal and/or misfolded proteins are prevented by three different mechanisms: the ubiquitin–proteasome system (UPS), the autophagy–lysosome pathway (ALP), and the interaction of molecular chaperones with UPS and ALP. These mechanisms regulate and prevent the accumulation of toxic proteins by breaking them into smaller polypeptide chains. The dysfunction of these cellular protein quality control systems causes the accumulation of misfolded proteins in neurons promoting neurodegeneration as observed also for AD [10,11,12,13,14]. Growing clinical and preclinical evidence, indeed, reveals that there is a relationship between the altered degradation of misfolded proteins, such as amyloid β (Aβ) peptides and hyperphosphorylated tau protein, and the pathogenesis of AD [15,16]. Ubiquitinated proteins have been found to be highly present in AD brain areas, including areas outside the forebrain, suggesting a direct link between the accumulation of misfolded proteins and a reduction in the clearance systems [17].

Brains from AD patients show distinct histopathological hallmarks which are extracellular deposits of Aβ peptides in the form of senile plaques, Aβ deposits in the cerebral blood vessels, and intracellular inclusion of neurofibrillary tangles (NFTs) composed of hyperphosphorylated tau protein [18]. Regarding the formation of Aβ deposits and NFTs, findings concerning which comes first are discordant; however, growing evidence recognizes the very early onset of tau pathology and its key role in mediating Aβ toxicity [19,20,21,22]. It was therefore suggested that total and phosphorylated tau accumulation may be pathologically more relevant than Aβ plaques to the development of neurodegeneration and cognitive decline in AD patients. Moreover, in the cerebrospinal fluid (CSF), tau levels correlate well with the formation of NFTs and can be used as a biomarker of Alzheimer-type pathological changes in the brain [23]. Nevertheless, although the involvement of tau in AD pathogenesis is well evident, the mechanisms leading to its accumulation are still not completely clear.

In normal conditions, tau stabilizes microtubules within axons and facilitates the axonal transport of neurotransmitters and other cellular molecules; in addition, it takes part to synaptic function and neuronal signaling [24,25]. Various post-translational modifications, including phosphorylation, truncation, nitration, glycosylation, and oxidation, occur in tau protein [26,27]; tau can also be acetylated, affecting its stability, and leading to its pathological transformation [28]. In particular, the hyperphosphorylation of tau reduces its binding to the microtubules [29], whereas tau acetylation inhibits the ubiquitin-mediated proteolysis of phosphorylated tau, thereby promoting its pathological aggregation in toxic insoluble oligomers [30]. Indeed, the tau oligomerization process may be further facilitated as tau proteins are dissociated from microtubules. The dysfunction of UPS and/or ALP is responsible for the accumulation inside neurons of insoluble NFTs, leading to neurodegeneration by impairing axonal transport and neuronal function [24].

In the last few years, a close link between brain cholesterol dyshomeostasis and AD pathogenesis has been recognized, and it is now believed that one of the main triggers in AD is oxidized cholesterol in the form of oxysterols [31]. Oxysterols, unlike cholesterol, diffuse across the blood–brain barrier (BBB) into the systemic circulation or from the systemic circulation into the brain [32], also due to damaged BBB integrity [33]. 

In relation to this, in a previous study, we clarified the association between oxysterol concentrations in the brain and the progression of the disease. Of note, the study underlined the significant reduction in 24-hydroxycholesterol (24-OHC) levels in the frontal and occipital cortices during the progression of AD; conversely, other oxysterols of enzymatic origin (e.g., 27-hydroxycholesterol) or derived from cholesterol autooxidation (e.g., 7-ketocholesterol) significantly increased. Moreover, in parallel with the loss of 24-OHC, the levels of the deacetylase sirtuin 1 (SIRT1) drastically decreased with the progression of the disease. SIRT1 reduction also correlated with neuroinflammation: increased inflammatory molecule levels (e.g., interleukins and chemokines) have been detected in the brain of AD patients in the earlier phases of the disease, precisely when SIRT1 levels begin to shrink [34].

SIRT1 is a NAD^+^-dependent protein deacetylase and takes part in many cellular processes by deacetylating protein substrates in various signal transduction pathways [35,36]. Of note, SIRT1 plays a crucial role in regulating neuron survival because of its several beneficial effects, including the ability to inhibit NFT and Aβ plaque formation [37,38].

It is likely that both 24-OHC and SIRT1 loss are factors in accelerating AD development. Indeed, the emerging idea is that 24-OHC could act in the brain by stimulating neuroprotective pathways such as the SIRT1-dependent pathways, as demonstrated in a recent study by our group [39]. The data obtained in vitro indicate that 24-OHC, by inducing the intracellular generation of reactive oxygen species (ROS), activates the SIRT1-dependent neuroprotective pathway to favor tau deacetylation and prevent intracellular accumulation of hyperphosphorylated tau. The neuroprotective action of 24-OHC was also shown to be effective against tau hyperphosphorylation in vivo, in hTau mice (carrying human tau), through an intracerebroventricular (ICV) injection of the oxysterol [39]. 

One of the downstream targets of SIRT1 is the transcriptional factor peroxisome proliferator-activated receptor gamma coactivator 1α (PGC1α), which acts as a transcriptional co-activator in the regulation of multiple signal transduction pathways affecting cell survival or death. It influences mitochondria respiration, antioxidant defense systems, and fatty acid metabolism by interacting with specific different transcription factors [40,41]; moreover, it is an important negative regulator of inflammatory mediator activation [42]. PGC1α, for example, can act as coactivator of nuclear factor erythroid 2-related factor 2 (Nrf2), also known as NFE2L2, another downstream transcriptional factor of SIRT1, by increasing its expression and antioxidant activity [43]; in turn, Nrf2 might regulate the activity of PGC1α [44].

Nrf2 normally resides in the cytoplasm and is tightly regulated; it is mainly controlled by four E3 ubiquitin ligase complexes which mediate its ubiquitynation and proteasomal degradation. Among them, Nrf2 interacts with Kelch-like ECH-associated protein 1 (KEAP1)-Cullin 3 (Cul3)-RING-box protein 1 (RBX) [45]. Under oxidative stress, the KEAP1-Cul3 ubiquitination system is disrupted as a result of an interruption in critical cysteine residues of KEAP1 [46]. This allows Nrf2 to translocate into the nucleus where it binds to the antioxidant response elements (ARE) and regulates the transcription of several antioxidant, detoxifying, and anti-inflammatory genes, as well as genes encoding for proteins involved in the regulation of autophagy and clearance of damaged proteins, such as the proteasomal subunits. Indeed, Nrf2 not only orchestrates endogenous antioxidant enzymatic defenses but also enhances the degradation of oxidatively damaged proteins, by regulating proteasomal activity, pointing out the existence of an interaction between proteasomal activity and redox function [47,48,49,50]. Because of its several transcriptional functions, Nrf2-ARE binding is of great significance in neuroprotection [51]. 

Several studies have demonstrated that, by regulating acetylation and activity level of PGC1α, SIRT1 also deacetylates Nrf2, thus increasing its stability and promoting its translocation into the nucleus and thus the induction of Nrf2 transcriptional activity. These regulatory interactions suggest that SIRT1 is able to indirectly affect various cellular antioxidant defense mechanisms to promote protection when the cells are exposed to oxidative stress [36,52,53]. Of note, favoring the interaction between PGC1α and Nrf2, both transcriptional regulators might synergistically participate in the regulation of the UPS. 

In the present study we aimed at investigating, in SK-N-BE neuroblastoma cells, whether 24-OHC might modulate the SIRT1/PGC1α/Nrf2 signaling pathway by promoting tau ubiquitination and subsequently its proteolytic degradation by UPS, thus preventing intraneuronal tau accumulation. Moreover, to confirm the potential role of the SIRT1/PGC1α/Nrf2 axis in neuroprotection, we aimed at investigating gene expression and protein levels of the single molecules in the frontal and occipital cortices of AD patients. 

The results could reveal, for the first time, a beneficial role of 24-OHC in reducing tau accumulation in the SK-N-BE neuroblastoma cells by acting on the SIRT1/PGC1α/Nrf2 signaling pathway leading to ubiquitination and degradation of tau through UPS. A reduction in the protein levels of the three molecules involved in the signaling pathway was observed in AD brains. Of note, a marked reduction in SIRT1 expression was also observed in AD brains, while the expression of both PGC1α and Nrf2 were increased; this could be explained by the fact that their expression might be newly induced by oxidative stress and inflammation, as an adaptive response to neuropathological changes in AD [54]. 

These data highlight the importance of preventing the loss of 24-OHC in the AD brain, which was demonstrated in our previous study [34], in order to preserve the activity of this pathway and, consequently, to limit tau accumulation during the progression of AD.

## 2. Materials and Methods

### 2.1. Autopsy Specimens of AD Brains

Autopsy specimens from the frontal and occipital cortex of 14 AD patients (Early AD: 6 samples; Late AD: 8 samples) and 9 control subjects were provided by the Division of Neurology 5 and Neuropathology, Fondazione IRCCS Istituto Neurologico Carlo Besta (Milan, Italy). The cortex specimens were classified based on the Braak stage of neurofibrillary pathology [55] and the Thal stage based on Aβ deposits [56]. 

In the brain samples included in the present study (healthy and AD brains), a routine neuropathological examination excluded relevant lesions such as tumors, significant vascular disease/stroke, or inflammation. Specimens were assessed histologically by hematoxylin and eosin, cresyl violet for Nissl substance, Heidenhein-Woelcke for myelin, and thioflavine S for amyloid, as well as by immunohistochemistry using antibodies against Aβ (4 G8 1:4000, Signet Laboratories, Dedham, MA, USA) after formic acid pre-treatment for 30 min, and against phosphorylated tau (p-tau) (AT8 1:300, Innogenetics, Ghent, Belgium). The immunoreaction was visualized using the EnVision Plus/Horseradish Peroxidase system (DakoCytomation) and 3,3′- diaminobenzidine as chromogen.

### 2.2. Cell Culture and Treatments

SK-N-BE neuroblastoma cells were maintained in RPMI 1640 medium supplemented with 10% fetal bovine serum (EuroClone, Pero, Italy), 100 U/mL penicillin, 100 μg/mL streptomycin, and 2 mM glutamine (Sigma-Aldrich, Merck-Millipore, Darmstadt, Germany) at 37 °C with 5% CO_2_ in humidified atmosphere. Cells were treated with 1 μM 24-OHC (Avanti Polar Lipids Inc., Alabaster, AL, USA) dissolved in ethanol (0.1% *v*/*v*). In some experiments, cells were pre-treated for 3 h with 10 µM SR-18292 (Sigma-Aldrich, Merck-Millipore), an inhibitor of PGC1α, or co-treated with 0.1 µM MG-132 (Selleckchem, Houston, TX, USA), an inhibitor of proteasome activity, both dissolved in dimethyl sulfoxide (DMSO, 0.5% or 0.1% *v*/*v*, respectively), and then treated with the oxysterol. Final inhibitor concentrations were not cytotoxic (Figure S1). Incubation times for all experiments are reported in the Section 3 and/or Figure legends. 

### 2.3. RNA Extraction, cDNA Synthesis, and Real-Time RT-PCR

Total RNA was extracted using TriFast Reagent (Eurogold TriFast, EuroClone) following the manufacturer’s instructions. RNA was dissolved in RNase-free water fortified with RNase inhibitors (RNaseSUPERase-In RNase inhibitor, Invitrogen, Thermo Fisher Scientific, Waltham, MA, USA). The amount and purity (A260/A280 ratio) of the extracted RNA were assessed spectrophotometrically. cDNA was synthesized by reverse transcription from 2 μg RNA with a commercial kit and random primers (High-Capacity cDNA Reverse Transcription Kit, Applied Biosystems, Thermo Fisher Scientific) following the manufacturer’s instructions.

Singleplex real-time RT-PCR was performed with a 7500 Fast Real-Time PCR System (Applied Biosystems, Thermo Fisher Scientific) on 30 ng cDNA using TaqMan Gene Expression Assay kits prepared for human PGC1α (Hs01016719_m1), Nrf2 (Hs00975961_g1), SIRT1 (Hs01009006_m1), heme oxygenase 1 (HO-1) (Hs01110250_m1), NAD(P)H:quinone oxidoreductase-1 (NQO1) (Hs00168547_m1), and β_2_-microglobulin (Hs00187842_m1), and TaqMan Fast Universal PCR Master Mix (Applied Biosystems, Thermo Fisher Scientific). The oligonucleotide sequences are not revealed by the manufacturer because of proprietary interests. The cycling parameters have been described previously [34]. The fractional cycle number (Ct) at which fluorescence passes the threshold in the amplification plot of fluorescence signal versus cycle number was determined for each gene considered. The results were then normalized to the expression of β_2_-microglobulin. Relative quantification of target gene expression was achieved with a mathematical method [57].

### 2.4. Protein Extraction and Western Blotting 

SK-N-BE cells were lysed in ice-cold lysing buffer (1 mL PBS fortified with 10 μL Triton X-100, 10 μL 10% SDS, 5 μL DTT 1 M, 6 μL 0.1% PMSF, and 10 μL aprotinin) for 30 min, sonicated, and then centrifuged at 18,620× *g* for 15 min at 4 °C. Fresh frozen brains were homogenized in ice-cold RIPA-buffer (20 mM TRIS-HCl pH 7.4, 150 mM NaCl, 5 mM EDTA, 1% Triton X-100, phosphatase and protease inhibitors) (1 mL buffer/100 mg tissue), and then centrifuged at 18,620× *g* for 30 min at 4 °C. Total protein content was spectrophotometrically measured by Bradford’s method [58].

To analyze the protein levels, 50 μg (cell lysates) or 30 μg (brain lysates) of total proteins were separated by electrophoresis in 10% precast gels (TGX Stain-Free FastCast Acrylamide kit, Bio-Rad Laboratories Inc., Hercules, CA, USA), then transferred to nitrocellulose membranes (Amersham Protran, GE Healthcare, Chicago, IL, USA). 

To analyze acetyl-PGC1α, Nrf2, acetyl-Nrf2, Nrf2/KEAP1, and Nrf2/Cul3 complexes, ubiquitinated and total tau levels in cell lysates and PGC1α levels in brain lysates, 60–80 μg of total proteins, in RIPA-buffer with protease inhibitors, were immunoprecipitated with 2 μL of anti-PGC1α, anti-Nrf2, or anti-tau primary antibodies and incubated overnight at 4 °C. Then, 15 μL of protein A-Sepharose resin was added and the samples were incubated for 2 h at 4 °C and then centrifuged at 590× *g* for 5 min. Proteins were separated by electrophoresis and transferred to nitrocellulose membranes. After saturation of non-specific binding sites with 5% non-fat milk in Tris-buffered saline (TBS) 1X-Tween 20 0.1% for 1 h, at room temperature, the membranes were immunoblotted overnight at 4 °C with specific primary antibodies. We used primary antibodies against PGC1α (1:1000), Nrf2 (1:1000), KEAP1 (1:1000), Cul3 (1:1000), ubiquitin (1:1000) (Immunological Sciences, Roma, Italy), acetyl-Lys (1:1000) (Cell Signaling Technology, Denver, CO, USA), tau (1:4000) (Dako, Agilent Technologies, Santa Clara, CA, USA), or against SIRT1 (1:1000) (Merck-Millipore). After washing to remove unbound primary antibodies with TBS1X-Tween 20 0.1%, the membranes were probed with anti-mouse (1:2000) or anti-rabbit (1:2000) (Cell Signaling Technology) secondary antibodies for 1 h at room temperature. Some membranes were stripped and re-immunoblotted with anti-β-actin (1:1000) primary antibody (Sigma-Aldrich, Merck-Millipore) and then with anti-mouse secondary antibody (1:2000) (Cell Signaling Technology). The immunoreactive bands were visualized through enhanced chemiluminescence using Clarity Western ECL Substrate (Bio-Rad Laboratories Inc.) following the manufacturer’s protocol and acquired by ChemiDoc Imaging System (Bio-Rad Laboratories Inc). The bands were quantified by densitometric analysis using the Image J 1.43 software. When possible, the results were evaluated as relative units determined by normalization of the density of each band to that of the corresponding β-actin protein.

### 2.5. Immunofluorescence Confocal Microscopy

Cells were grown on glass slides into 6-well plates. After treatments, cells were washed with 0.1 M PBS, fixed in 4% formalin for 15 min at room temperature, and then washed again twice with PBS. After blocking non-specific binding sites with 0.1 M PBS containing 5% goat serum, 3% BSA, and 0.3% Tween 20, for 30 min, slides were incubated in the presence of primary antibodies against Nrf2 (1:150) (Immunological Sciences) or against tau (1:200) (Dako, Agilent Technologies) overnight at 4 °C; then, they were incubated for 1 h at room temperature with the secondary antibodies conjugated with the fluorescent probes Alexa Fluor 488 (1:400) (Immunological Sciences) or Alexa Fluor 594 (1:200) (Invitrogen), respectively. Nuclei were stained with propidium iodide (PI) (10 µg/mL) or Hoescht 33258 (10 µg/mL) (Sigma-Aldrich, Merck-Millipore). Slides, mounted with Fluoroshield (Sigma-Aldrich, Merck-Millipore), were observed by a LSM 800 confocal laser microscope (Carl Zeiss S.p.A, Oberkochen, Germany) equipped with a Plan-Apochromat 63x/1.40 DIC oil immersion objective. The wavelengths for each probe are Alexa Fluor 488, 490/525 nm (excitation/emission); Alexa Fluor 594, 590/617 nm (excitation/emission); PI, 535/617 nm (excitation/emission); Hoescht 33258, 352/461 nm (excitation/emission). The images were processed using Fiji Image J 1.53f51 software.

### 2.6. Small Interfering RNA Transfection

SIRT1 and Nrf2 small interfering RNAs (siRNAs) were used for transient gene knockdown studies (S223591 and S9493, respectively, Ambion, Thermo Fisher Scientific). Transfection of SIRT1 and Nrf2 siRNAs was performed following the manufacturer’s instructions. A non-targeting siRNA (scrambled siRNA) was used as a negative control (Silencer Select Negative Control #1 siRNA, Ambion, Thermo Fisher Scientific). Briefly, SK-N-BE cells were seeded into 24-well plates (4 × 10^4^ cells/500 μL) and 25 μL of 100 nM siRNA (SIRT1 or Nrf2) were mixed with equal volume (1:1 *v*/*v*) of transfection reagent solution containing 1.5 μL of transfection reagent (Lipofectamine^®^ RNAiMAX Reagent, Invitrogen) and left for 10 min in RPMI medium with 1% FBS and without antibiotics. After reverse transfection with SIRT1 siRNA or with Nrf2 siRNA (6 h), the medium was changed with fresh medium; cells were then incubated with 1 μM 24-OHC for 16 h or for 24 h, respectively. The silencing efficiency, validated by real-time RT-PCR, was approximately 77% for SIRT1 and 84% for Nrf2 (Figure S2). 

### 2.7. Proteasome Activity Assay

Proteasomal chymotrypsin-like activity was analyzed using a Proteasome Activity Assay Kit (ab107921, Abcam, Cambridge, UK) which utilizes a peptide substrate containing a highly fluorescent 7-amino-4-methyl coumarin (AMC) group (Succ-LLVY-AMC). The proteolytic activity was measured by AMC fluorescence release. Briefly, SK-N-BE cells were seeded into 6-well plates (2 × 10^5^ cells/2 mL), transfected with Nrf2 siRNA as described above, and then treated with 1 μM 24-OHC for 24 h; cells were then lysed in 0.5% NP-40 in PBS 1X and centrifuged at 16,080× *g* at 4 °C for 10 min. Cell lysate (15 μg) was incubated with the proteasomal substrate at 37 °C for 25 min. Fluorescence was measured by a fluorometric microplate reader (Infinite 200, Tecan, Männedorf, Switzerland) at 360/465 nm (excitation/emission). Proteasome activity, expressed as percentage of control value (untreated cells), was calculated following the manufacturer’s protocol. 

### 2.8. Cell Viability Assay

Cells were seeded in 96-well plates (2 × 10^4^ cells/100 μL) and incubated with SR-18292 or MG-132 inhibitors, at different concentrations for 24 h. Cell viability was analyzed by the MTT assay. This colorimetric assay is based on the reduction of the yellow tetrazolium salt 3-(4,5-dimethylthiazol-2-yl)-2,5-diphenyltetrazolium bromide (MTT) to purple formazan crystals by metabolically active cells; the viable cells contain NAD(P)H-dependent oxidoreductase enzymes which reduce the MTT to formazan. After cell treatments, 10 μL of 12 mM MTT was added and cells were kept in the dark for 90 min at 37 °C. The insoluble formazan crystals were dissolved replacing the medium with DMSO (120 μL). After 15 min, the resulted colored solution was quantified using a multi-well plate reader (Model 680 Microplate Reader, Bio-Rad) measuring the absorbance at 550 nm. Cell viability was expressed as percentage of control value (cells incubated with DMSO).

### 2.9. Statistical Analysis

All values are expressed as means ± SD. Data were analyzed statistically using one-way ANOVA with Bonferroni’s post-test for multiple comparisons or Student’s t test. Differences at *p* < 0.05 were considered statistically significant. Calculations were performed using GraphPad Prism 7 software (GraphPad Software Inc., La Jolla, CA, USA).

## 3. Results

### 3.1. Over-Expression and Deacetylation of PGC1α by 24-OHC

In a previous study, we demonstrated that 24-OHC is able to up-regulate the deacetylase SIRT1 [39]. 

**Figure 1:**
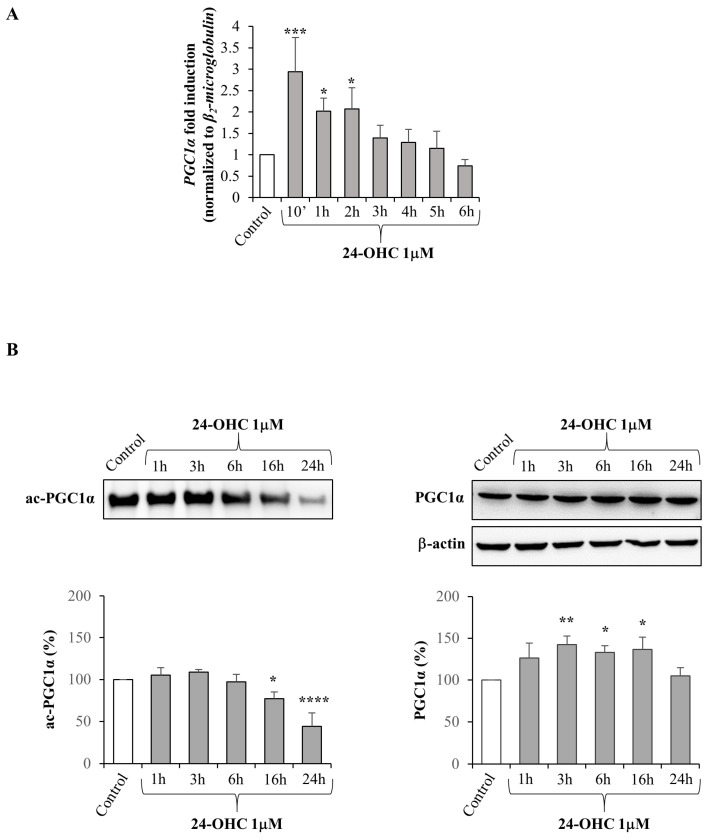
Effect of 24-OHC on PGC1α expression and on the levels of acetylated and total PGC1α in SK-N-BE cells. (**A**) PGC1α gene expression was quantified by real-time RT-PCR in cells treated with 24-OHC (1 µM) up to 6 h. Data, normalized to the corresponding β_2_-microglobulin, are expressed as mean values ± SD of three different experiments. * *p* < 0.05 and *** *p* < 0.001 vs. control. (**B**) Acetylated PGC1α (ac-PGC1α) and total PGC1α protein levels were analyzed by Western blotting in cells treated with 1 µM 24-OHC up to 24 h. Representative blots are shown. The histograms represent mean values ± SD of three experiments. Total PGC1α levels were normalized to the corresponding β-actin levels. Densitometric measurements are expressed as percentage of control value. * *p* < 0.05, ** *p* < 0.01, **** *p* < 0.0001 vs. control.

With the aim of investigating whether 24-OHC might modulate the expression of PGC1α, a well-known downstream target of SIRT1, SK-N-BE neuroblastoma cells were incubated up to 6 h with the oxysterol at the physio-pathological concentration of 1 μM. PGC1α gene expression was found to be significantly up-regulated by 24-OHC already after 10 min; after 2 h cell treatment there was a progressive reduction in PGC1α expression levels until control values were reached at 6 h (Figure 1A). 

It is known that PGC1α can be deacetylated by SIRT1, and PCG1α deacetylation is required for the activation of different signal pathways, including the Nrf2 pathway [36,52,53]. 

On this basis, we analyzed the ability of 24-OHC to induce, presumably through SIRT1 up-regulation, PGC1α deacetylation. In SK-N-BE cells treated with the oxysterol (1 μM), it emerged that the levels of acetylated PGC1α (ac-PGC1α) were significantly decreased from 16 h to 24 h underlying the increase in transcriptional coactivator deacetylation. Levels of total PGC1α were also measured and a significant increase in its levels was shown at almost all times considered after the 24-OHC treatment (Figure 1B).

### 3.2. 24-OHC Regulates the Expression of Nrf2 and, through SIRT1, Its Deacetylation

**Figure 2:**
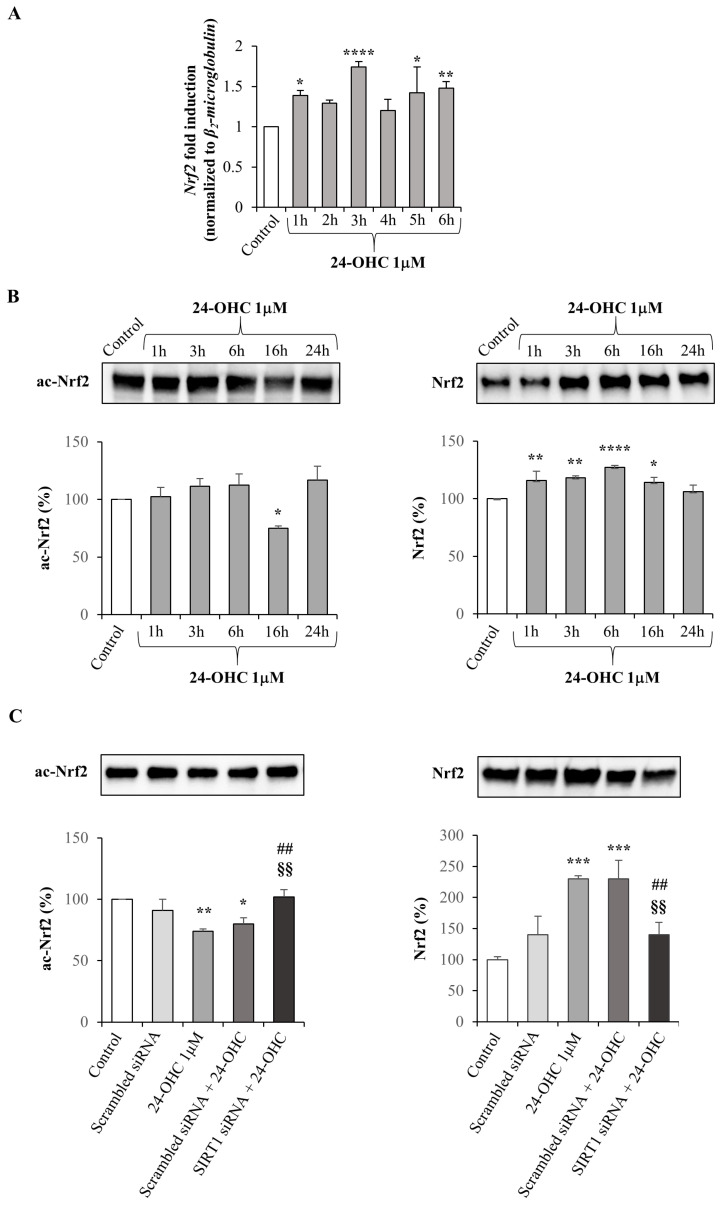
Effect of 24-OHC on Nrf2 expression and on the levels of acetylated and total Nrf2, and SIRT1-dependent Nrf2 acetylation in SK-N-BE cells. (**A**) Nrf2 gene expression was quantified by real-time RT-PCR in cells treated with 24-OHC (1 µM) up to 6 h. Data, normalized to the corresponding β_2_-microglobulin, are expressed as mean values ± SD of three different experiments. * *p* < 0.05, ** *p* < 0.01, **** *p* < 0.0001 vs. control. (**B**) Acetylated Nrf2 (ac-Nrf2) and total Nrf2 protein levels were analyzed by Western blotting in cells treated with 24-OHC (1 µM) up to 24 h. Representative blots are shown. The histograms represent mean values ± SD of three experiments. Densitometric measurements are expressed as percentage of control value. * *p* < 0.05, ** *p* < 0.01, **** *p* < 0.0001 vs. control. (**C**) Acetylated Nrf2 (ac-Nrf2) and total Nrf2 protein levels were analyzed in cells transfected for 6 h with SIRT1 or scrambled siRNA and then treated with 24-OHC (1 µM) for 16 h. Representative blots are shown. The histograms represent mean values ± SD of three experiments. Densitometric measurements are expressed as percentage of control value. * *p* < 0.05, ** *p* < 0.01, *** *p* < 0.001 vs. control; ## *p* < 0.01 vs. 24-OHC; §§ *p* < 0.01 vs. scrambled siRNA + 24-OHC.

To check whether 24-OHC may also up-regulate the transcriptional factor Nrf2, SK-N-BE cells were incubated with 1 μM 24-OHC up to 6 h. Nrf2 expression was increased from 1 h to 6 h with a maximum at 3 h (Figure 2A). 

To demonstrate that Nrf2 is also a downstream target of SIRT1, both acetylated Nrf2 (ac-Nrf2) and total Nrf2 levels were analyzed by Western blotting. A significant decrease in ac-Nrf2 levels occurred only after 16 h cell treatment, compared to untreated cells (control) while the total levels of Nrf2 were significantly higher than control from 1 h up to 16 h (Figure 2B). 

The decreased acetylation of Nrf2 at 16 h resulted from SIRT1 activation by 24-OHC, as demonstrated by using a specific SIRT1 siRNA: when SIRT1 was silenced the levels of ac-Nrf2 markedly increased. The total levels of Nrf2 were higher than the control in cells treated with 24-OHC but were significantly decreased when SIRT1 was silenced (Figure 2C). 

These results point out that SIRT1 is involved in both acetylation and synthesis of Nrf2.

### 3.3. 24-OHC Promotes the Nuclear Translocation of Nrf2 and Activation of its Antioxidant Target Genes

As demonstrated above, SIRT1 can deacetylate Nrf2; consequently, this promotes Nrf2 translocation into the nucleus where it binds to ARE which mediate the transcriptional activation of cytoprotective genes, including genes involved in antioxidant defenses [47,48,49,50].

**Figure 3:**
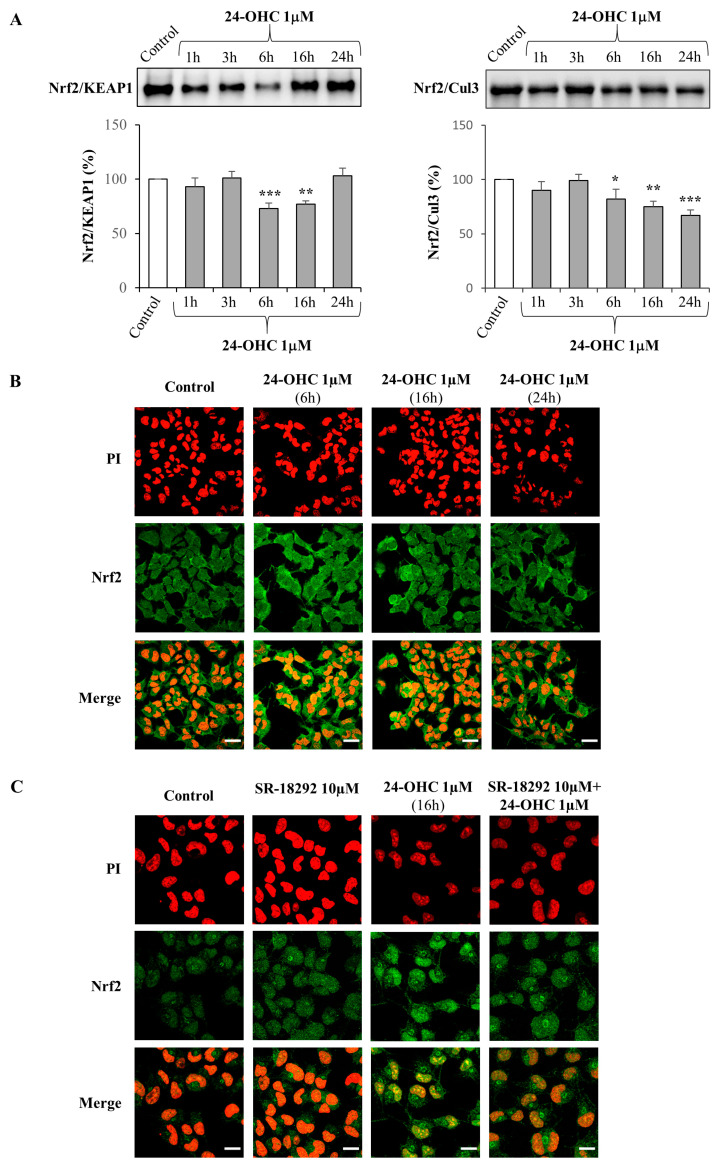
Effect of 24-OHC on Nrf2/KEAP1 and Nrf2/Cul3 complexes and nuclear translocation of Nrf2 in SK-N-BE neuroblastoma cells. Inhibition of PGC1α prevents Nrf2 nuclear translocation. (**A**) Both Nrf2/KEAP1 and Nrf2/Cul3 complexes were analyzed by Western blotting in cells treated with 1 µM 24-OHC up to 24 h. Representative blots are shown. The histograms represent mean values ± SD of three experiments. Densitometric measurements are expressed as percentage of control value. * *p* < 0.05, ** *p* < 0.01, *** *p* < 0.001 vs. control. (**B**) Cells were treated with 1 µM 24-OHC for 6, 16, and 24 h or (**C**) cells were pre-treated (3 h) with 10 µM SR-18292 and then treated with 24-OHC for 16 h. Nrf2 nuclear translocation was detected by confocal laser microscopy equipped with a Plan-Apochromat 63x/1.40 DIC oil immersion objective. Nuclei were stained with PI (exciting max/emission max: 535 nm/617 nm) and Nrf2 was revealed by a fluorescent probe (Alexa Fluor 488, exciting max/emission max: 490 nm/525 nm). The images are representative of two experiments ((**B**) scale bar 30 µm; (**C**) scale bar 15 µm).

To prove that, SK-N-BE cells were incubated with 24-OHC (1 μM) from 1 h to 24 h and both Nrf2/KEAP1 and Nrf2/Cul3 complexes were analyzed. Concerning the Nrf2/KEAP1 complex, the dissociation, due to a conformation change of KEAP1, was significantly observed after 6 h and 16 h cell treatment, as shown by the lower amount of the complex; for the Nrf2/Cul3 complex, the dissociation was observed from 6 h up to 24 h cell treatment (Figure 3A). As a consequence of the repressor KEAP1 and Cul3 dissociation, Nrf2 translocated into the nucleus as detected by laser confocal microscopy: a marked nuclear translocation of Nrf2 was present already after 6 h and up to 24 h of cell treatment with 24-OHC (Figure 3B). 

To demonstrate that PGC1α, activated by 24-OHC, might influence the nuclear translocation of Nrf2, cells were pre-incubated (3 h) with 10 µM SR-18292, an inhibitor of PGC1α, and then treated for 16 h with the oxysterol: again, 24-OHC clearly induced Nrf2 nuclear translocation, which was partially inhibited by the presence of the PGC1α inhibitor (Figure 3C).

**Figure 4:**
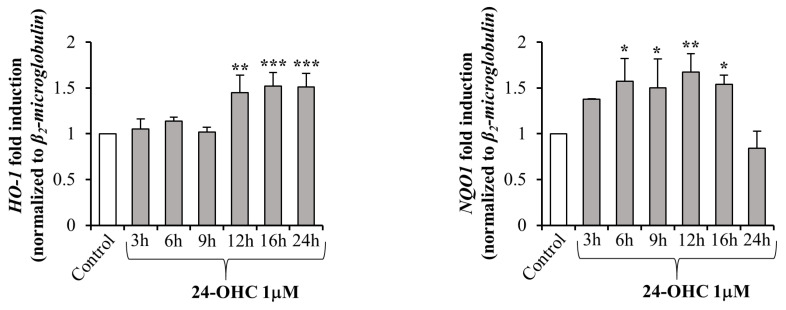
Nrf2-target gene expression. HO-1 and NQO1 expression was quantified by real-time RT-PCR in cells treated with 1 µM 24-OHC up to 24 h. Data, normalized to the corresponding β_2_-microglobulin, are expressed as mean values ± SD of 3 different experiments. * *p* < 0.05, ** *p* < 0.01, *** *p* < 0.001 vs. control.

Finally, to prove the transcriptional activity of Nrf2, two antioxidant target genes of the transcription factor were analyzed: HO-1 and NQO1. HO-1 expression levels were significantly increased after 12 h up to 24 h cell treatment with 24-OHC, while NQO1 expression was significantly increased between 6 h and 16 h of cell treatment (Figure 4).

### 3.4. 24-OHC Induces Tau Protein Ubiquitination through Nrf2 Activation

We have previously shown in vitro and in vivo that 1 µM 24-OHC is able to increase the deacetylation of tau and to reduce the levels of phosphorylated tau through SIRT1 up-regulation [39]. 

After demonstrating the SIRT1/PGC1α/Nrf2 interaction, we hypothesized that 24-OHC, through this signal pathway, induces tau protein deacetylation, ubiquitination, and then its degradation via proteasome. 

**Figure 5:**
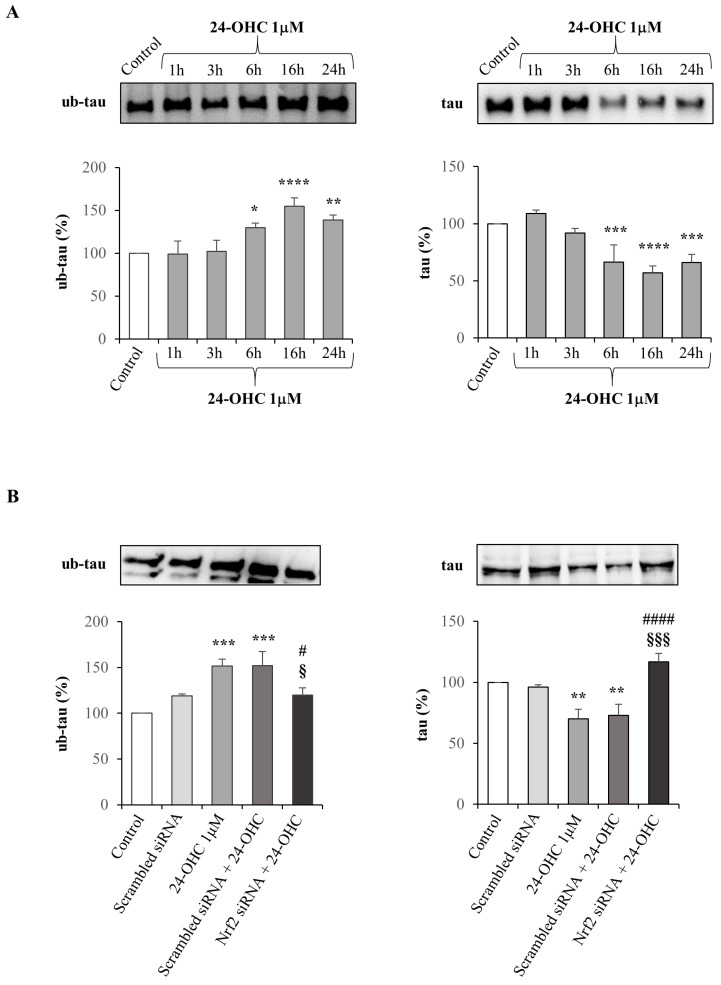
Effect of 24-OHC on the levels of ubiquitinated and total tau, and Nrf2-dependent tau ubiquitination in SK-N-BE cells. (**A**) Ubiquitinated tau (ub-tau) and total tau protein levels were analyzed by Western blotting in cells treated with 24-OHC up to 24 h. Representative blots are shown. The histograms represent mean values ± SD of three experiments. Densitometric measurements are expressed as percentage of control value. * *p* < 0.05, ** *p* < 0.01, *** *p* < 0.001, **** *p* < 0.0001 vs. control. (**B**) Ubiquitinated tau (ub-tau) and total tau protein levels were analyzed in cells transfected for 6 h with Nrf2 or scrambled siRNA and then treated with 24-OHC (1 µM) for 24 h. Representative blots are shown. The histograms represent mean values ± SD of three experiments. Densitometric measurements are expressed as a percentage of the control value. ** *p* < 0.01, *** *p* < 0.001 vs. control; # *p* < 0.05, #### *p* < 0.0001 vs. 24-OHC; § *p* < 0.05, §§§ *p* < 0.001 vs. scrambled siRNA + 24-OHC.

SK-N-BE cells were incubated with 1 µM 24-OHC up to 24 h and the ubiquitinated tau (ub-tau) and total tau levels were analyzed by Western blotting. The levels of ub-tau increased significantly from 6 h up to 24 h cell treatment while in parallel the levels of total tau decreased. Concerning total tau, the reduction in the protein levels was presumably due to the contemporary tau proteasomal degradation (Figure 5A).

It was further demonstrated, using a specific Nrf2 siRNA, that the effect of the oxysterol, represented by the increase in tau ubiquitination and consequently in tau degradation, actually depends on the transcriptional activity of Nrf2. After Nrf2 silencing, the ubiquitination of tau was markedly reduced compared to non-transfected cells treated with 24-OHC for 24 h; on the contrary total tau levels were significantly increased when Nrf2 was silenced (Figure 5B).

### 3.5. Degradation of Tau by the Ubiquitin–Proteasome System

Among the mechanisms that prevent the accumulation in neurons of toxic proteins, such as hyperphosphorylated tau, the UPS was investigated in our study. 

**Figure 6:**
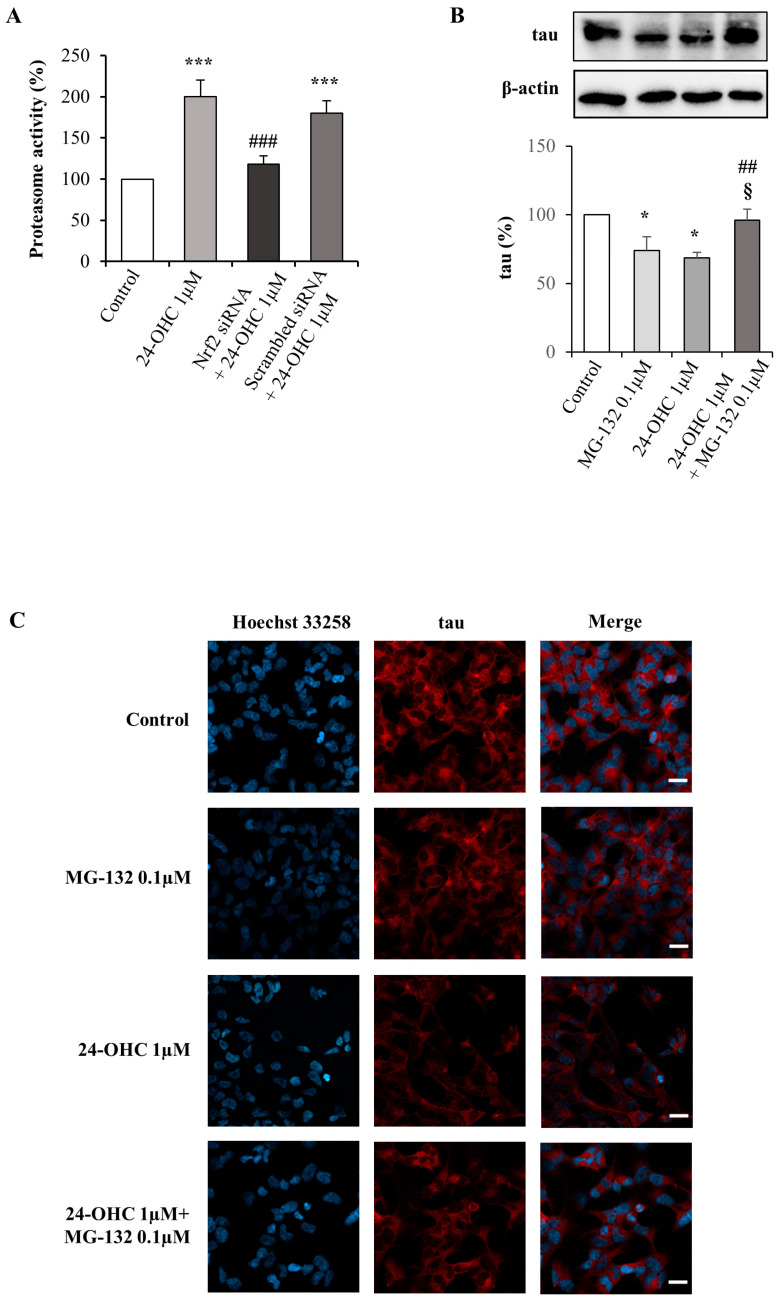
Effect of 24-OHC on Nrf2-dependent proteasome activity and on tau proteasomal degradation. (**A**) Proteasomal chymotrypsin-like activity was analyzed using a commercial kit. Cells were transfected for 6 h with Nrf2 or scrambled siRNA and then treated with 24-OHC (1 µM) for 24 h. The histograms represent mean values ± SD of three experiments and expressed as percentage of control value. *** *p* < 0.001 vs. control; ### *p* < 0.001 vs. 24-OHC. (**B**) Tau protein levels were analyzed by Western blotting in 24-OHC-treated cells for 24 h and co-treated or not with 0.1 µM MG-132, an inhibitor of proteasome activity. Representative blots are shown. The histograms represent mean values ± SD of three experiments. Tau densitometric measurements were normalized against the corresponding β-actin levels and expressed as percentage of control value. * *p* < 0.05 vs. control; ## *p* < 0.01 vs. 24-OHC; § *p* < 0.05 vs. MG-132. (**C**) After 24-OHC (1 µM) cell treatment (24 h), with or without MG-132 (0.1 µM), tau protein levels were detected by confocal laser microscopy equipped with a Plan-Apochromat 63x/1.40 DIC oil immersion objective, scale bar 20 µm. Nuclei were stained with Hoechst 33258 (exciting max/emission max: 352 nm/461 nm) and tau was revealed by a fluorescent probe (Alexa Fluor 594, exciting max/emission max: 590 nm/617 nm). The images are representative of two experiments.

SK-N-BE neuroblastoma cells, transfected or not with the specific Nrf2 siRNA, were incubated with 24-OHC (1 µM) and the proteasome activity was investigated using a commercial kit. Cell treatment with the oxysterol significantly increased the activity of the proteasome. Notably, it was markedly and significantly prevented when the Nrf2 was silenced (Figure 6A). The data underline the involvement of the transcriptional factor in the induction of the proteasome activity. 

To verify if tau is effectively degraded via proteasome, some cells were incubated with 24-OHC (24 h) with or without 0.1 µM MG-132 (co-incubation), a molecule specifically able to inhibit the proteasome degradation of ubiquitinated proteins. The amount of tau protein was significantly higher in cells co-treated with 24-OHC and MG-132 compared to cells treated only with 24-OHC or MG-132 (Figure 6B). Of note, tau reduction in the presence of MG-132 alone could suggest that during the 24 h treatment other degradative pathways, such as autophagy, might be induced in the cells to compensate UPS block. However, these mechanisms were unable to act on ubiquitinated tau, whose formation was promoted by 24-OHC, thus leading to protein accumulation when UPS activity was also inhibited. These results were further confirmed by immunofluorescence, using a laser confocal microscope, after 24 h of treatment with 24-OHC, with or without the proteasome inhibitor; the intracellular levels of tau were markedly increased in the cells co-treated with MG-132 (Figure 6C).

Overall, these data point out that UPS might be one of the main mechanisms, triggered by 24-OHC, to prevent the accumulation of hyperphosphorylated tau thus preventing NFT formation and neurodegeneration in AD brains.

### 3.6. Expression and Synthesis of SIRT1, PGC1α, and Nrf2 in AD Brains

With the aim to investigate the involvement of the SIRT1/PGC1α/Nrf2 pathway in neuroprotection, the gene expression and the protein levels of the single molecules were evaluated in the frontal and occipital cortex samples of AD brains, which were classified based on the Braak staging system of neurofibrillary pathology [55] and indicated as Early AD (stages I to III, age at death from 71 to 86 years; six samples) or Late AD (stages IV to VI, age at death from 75 to 83 years; eight samples). Brains of control subjects (age at death from 58 to 70 years; nine samples) were also examined. Detailed information about the subjects included in the analysis is reported in Table 1. 

**Figure 7:**
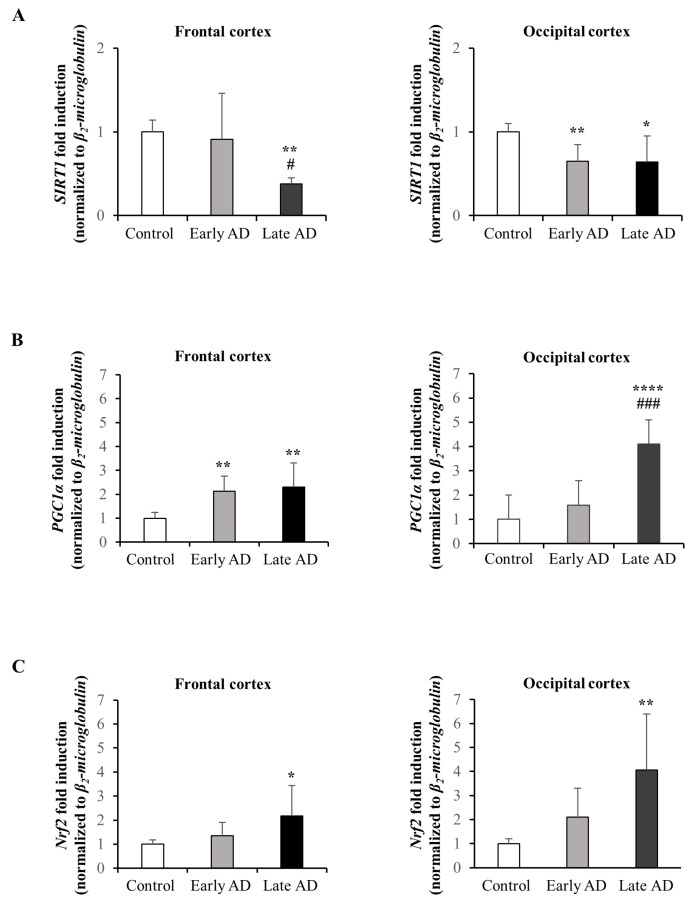
Gene expression of SIRT1 (**A**), PGC1α (**B**), and Nrf2 (**C**) in brain samples. Gene expression was quantified by real-time RT-PCR in all autopsy specimens from the frontal and occipital cortex of AD patients at different stages of neurofibrillary pathology (Early AD: *n* = 6; Late AD: *n* = 8) and healthy subjects (Control: *n* = 9). Data, normalized to the corresponding β_2_-microglobulin, are expressed as mean values ± SD. * *p* < 0.05, ** *p* < 0.01, **** *p* < 0.0001 vs. control; # *p* < 0.05, ### *p* < 0.001 vs. Early AD.

**Figure 8:**
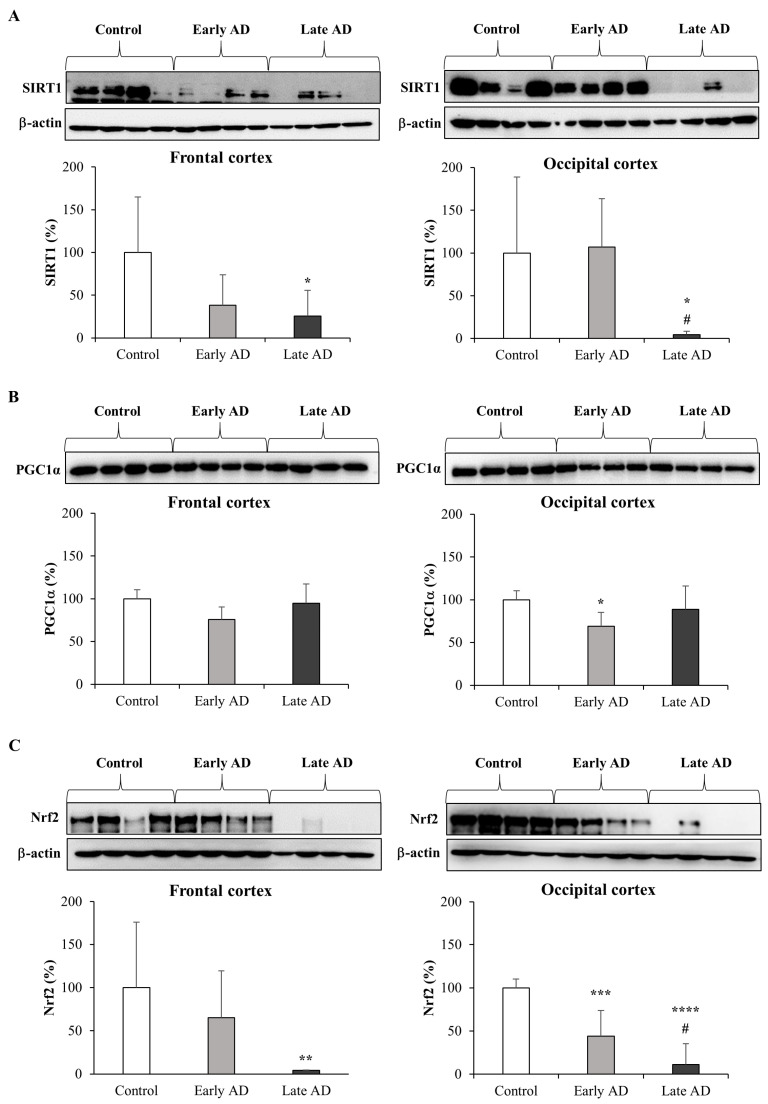
Protein levels of SIRT1 (**A**), PGC1α (**B**), and Nrf2 (**C**) in brain samples. Protein levels were analyzed by Western blotting in all frontal and occipital cortex specimens of AD (Early AD: *n* = 6; Late AD: *n* = 8) and of healthy subjects (Control: *n* = 9). Representative blots are shown. SIRT1 and Nrf2 densitometric measurements were normalized against the corresponding β-actin levels. SIRT1, PGC1α, and Nrf2 densitometric measurements of the protein levels of all AD specimens are expressed as percentage of control value. * *p* < 0.05, ** *p* < 0.01, *** *p* < 0.001, **** *p* < 0.0001 vs. control; # *p* < 0.05 vs. Early AD.

The expression of SIRT1 significantly decreased with the progression of the disease in both frontal and occipital cortices compared to the control brains (Figure 7A), in agreement with the results reported in our previous study [34]. The trend was confirmed by Western blotting analysis of SIRT1 protein levels; the protein levels of SIRT1 were dramatically decreased in the Late AD brains in both frontal and occipital cortices compared to the control brains but in the frontal cortex SIRT1 levels are also lower in the Early AD, although not significantly (Figure 8A). 

The expression of both PGC1α and Nrf2 in the brains was increased with the progression of AD (Figure 7B,C); this could be explained by the fact that the expression of these two transcription regulators might be newly stimulated as a compensation against oxidative stress and neuroinflammation, events that are thought to exacerbate the pathology of AD [54]. However, in contrast, the protein levels of Nrf2 were found markedly decreased in both frontal and occipital cortices of Early and Late AD brains compared to control brains, presumably as a consequence of post-transcriptional modifications (Figure 8C). Concerning PGC1α, the protein levels decreased only in the Early AD of the frontal and occipital cortex specimens (Figure 8B).

## 4. Discussion

Although many studies have been carried out on AD pathogenesis, the molecular mechanisms and the sequence of events leading to neurodegeneration are not yet fully known. However, a growing bulk of evidence suggests a link between altered cholesterol metabolism and AD, and it is now believed that oxysterols, namely the cholesterol oxidation products, are key actors in AD pathogenesis [31,59,60]. Many studies have therefore focused on understanding the molecular mechanisms exerted by oxysterols that accumulate in the AD brain, showing that they lead to neuron dysfunction and degeneration contributing to oxidative stress, neuroinflammation, amyloidogenesis, tau accumulation, and cell death [31,32,34,61,62,63]. However, it is emerging that the behavior of certain oxysterols, including the ones accumulating in the brain, is peculiar: they are double-edged compounds, showing both detrimental and beneficial properties at the same time, depending on the experimental models and concentrations, since they are able to trigger both death and survival signals within cells [64]. 

Among the various oxysterols involved in AD pathogenesis, the effects of 24-OHC are contrasting since the data indicate either damaging or protective activities of this oxysterol, as reviewed by Gamba and colleagues [65]. Although its role in AD is controversial and still debated, the idea is now emerging that this oxysterol, produced almost exclusively in the brain, mainly exerts a neuroprotective action. 

Physiologically, the role of 24-OHC is essential to maintain brain cholesterol homeostatic regulatory loop between astrocytes and neurons, which ensures cholesterol availability for neuronal uptake and the elimination of excess cholesterol [32,66,67]. Besides that, several studies have pointed out its beneficial effects against AD progression, for example inducing an adaptive response [68], suppressing Aβ accumulation in the brain and neurons [69,70,71,72,73], and regulating synaptic plasticity and functions in rat hippocampal neurons and slices through the potentiation of responses mediated by N-methyl-D-aspartate receptors (NMDARs) [74,75].

Despite this evidence, the relationship of 24-OHC with tau accumulation and NFT formation is not yet clear. In this regard, a beneficial effect of 24-OHC was demonstrated in SK-N-BE neuroblastoma cells by our group, due to its ability to up-regulate both expression and synthesis of SIRT1, and consequently to lead to tau deacetylation and to prevent its phosphorylation. The neuroprotective action of 24-OHC was strongly supported by in vivo evidence obtained following the ICV injection of the oxysterol in hTau mice that carry human tau and develop tau pathology only after Aβ monomer administration. The pretreatment (4 days) with 24-OHC completely prevented tau hyperphosphorylation induced by Aβ injection in the brain of hTau mice [39]. The data suggest that 24-OHC plays a key role in preventing the intracellular accumulation of insoluble hyperphosphorylated tau. 

These data are in agreement with the study of Burlot’s group. The effects of 24-OHC in preventing tau accumulation were investigated in THY-Tau22 mice, an animal model of AD-like tau pathology. In the hippocampus of these animals the levels of both 24-OHC and CYP46A1, the enzyme which converts cholesterol in 24-OHC, were proven to be lower than in control mice. Of note, the injection of an adeno-associated virus-CYP46A1 vector brought CYP46A1 and 24-OHC content back to control levels and the cognitive deficits were remedied, whereas tau hyperphosphorylation and gliosis were unaffected. The data indicate that CYP46A1, by restoring 24-OHC levels in the brain, may be considered a potential therapeutic target for tauopathies, including AD [76].

Taking into account these data, it is likely that 24-OHC loss could be a factor in accelerating the disease. In support of the fundamental role played by 24-OHC in preventing AD pathogenesis, it has been discovered that during the progression of the disease the levels of 24-OHC markedly decrease. A study conducted in our laboratory on the post-mortem human AD brains (frontal and occipital cortices) demonstrated that 24-OHC levels decrease during AD progression. The progressive loss of 24-OHC in the AD-affected brains co-occurs with the loss of the neuroprotective deacetylase SIRT1. A marked increase in several inflammatory mediators was also observed in parallel with 24-OHC and SIRT1 loss. In particular, elevated inflammatory molecule levels have been detected in the brain of AD patients in the earlier phases of the disease, precisely when SIRT1 levels began to shrink [34]. The loss of SIRT1 might, indeed, favor neuroinflammation and NFT formation contributing to neurodegeneration, since its reduction is closely associated with Aβ and tau accumulation in the cerebral cortex of AD patients [77].

Based on our previous study in which we demonstrated, in vitro and in vivo, the ability of 24-OHC to up-regulate SIRT1 and consequently to increase tau deacetylation preventing hyperphosphorylated tau accumulation [39], in the present study we focused our attention on the effect of 24-OHC on the neuroprotective SIRT1/PGC1α/Nrf2 pathway with the aim to investigate whether its modulation by the oxysterol might reduce the intracellular accumulation of tau favoring its degradation. In relation to this, it has been hypothesized that, by inducing SIRT1-dependent tau deacetylation, 24-OHC makes the protein more susceptible to ubiquitination and proteasomal degradation, leading to total tau reduction in neurons [30]. 

As regards PGC1α, among its several functions it can act as a co-activator of Nrf2 which is activated by oxidative stress [43]; in turn, Nrf2 might regulate the activity of PGC1α [44]. Following its translocation into the nucleus, Nrf2 binds to several target genes, including genes that encode detoxifying, antioxidant, and anti-inflammatory proteins as well as those involved in the clearance of damaged proteins, such as some proteasomal subunits and autophagy related-proteins [48,49,50]. In this connection, besides being a master regulator of ARE-activated gene expression [78], Nrf2 is currently emerging as a key component of the transduction machinery that maintains proteostasis (i.e., the homeostatic control of protein synthesis), as well as protein folding, trafficking, and degradation. Among them, Nrf2 can intercept emergency signals derived from misfolded protein accumulation in order to build a coordinated and perdurable transcriptional response. This is achieved thanks to the Nrf2-mediated control of the genes involved in the maintenance of proteasome and autophagy activities [79].

Given the interaction between PGC1α and Nrf2 and the involvement of Nrf2 in the regulation of the proteasome activity [47,80], we can hypothesize that PGC1α and Nrf2 might synergistically participate in the regulation of the UPS as downstream targets of SIRT1. Indeed, SIRT1, regulating the acetylation and activity of PGC1α, is also able to activate Nrf2 transcriptional activity that is likely of great significance in neuroprotection through its several cytoprotective functions [54,81,82,83]. On these bases, increasing Nrf2 activity, several neurodegenerative processes leading to synaptic degeneration and neuronal death could be avoided. Among them, there are oxidative stress, chronic inflammation, mitochondrial dysfunction, Aβ peptide accumulation, proteasome inhibition, and hyperphosphorylated tau protein aggregation. Conversely, down-regulation of SIRT1 expression significantly reduced Nrf2 protein levels [84,85].

In this connection, we investigated whether SK-N-BE cell treatment with 24-OHC was able to induce Nrf2 activation, through the modulation of the SIRT1/PGC1α/Nrf2 pathway, promoting tau ubiquitination and subsequently its proteolytic degradation, probably by UPS activity. 

Given that the induction of SIRT1 by 24-OHC is a key point that has been previously demonstrated [39], in the present study the first evidence was the up-regulation of gene expression and total protein levels of PGC1α; moreover, a significant deacetylation of this transcription co-activator was observed in cells treated with 24-OHC, presumably by the deacetylase SIRT1 (Figure 1). Concerning Nrf2, its expression was increased and the levels of acetylated Nrf2 were decreased after 16 h cell treatment. An accumulation of newly synthesized Nrf2 was also observed following cell incubation with 24-OHC (Figure 2A,B). A marked deacetylation of Nrf2, as a result of SIRT1 activation by 24-OHC, was demonstrated by using a specific SIRT1 siRNA: in the transfected cells the deacetylation of Nrf2 was prevented (Figure 2C). Following its deacetylation and the dissociation of both Nrf2/KEAP1 and Nrf2/Cul3 complexes, Nrf2 was demonstrated to translocate into the nucleus (Figure 3A,B) where it binds to ARE which mediate the transcriptional activation of cytoprotective genes, such as the antioxidant genes HO-1 and NQO1 (Figure 4). SK-N-BE cell pre-treatment with SR-18292 (10 µM), an inhibitor of PGC1α, clearly demonstrated that the activation of Nrf2 occurs through PGC1α induction triggered by cell treatment with 24-OHC (Figure 3C). 

The interaction between SIRT1 and Nrf2 has already been proven by previous studies. For example, mice treatment with a high concentration of Aβ_25–35_ impaired the learning-memory ability and disordered the structure of neurons and mitochondria. Moreover, it has been observed that high concentration of Aβ_25–35_ decreased the SIRT1/Nrf2 related antioxidant capacity and induced apoptosis. In contrast, in mice treated with low concentration of Aβ_25–35,_ superoxide dismutase 1 (SOD1) levels and SIRT1/Nrf2 expression were increased and autophagy was induced [86]. Moreover, the knockdown of SIRT1 has been shown to inhibit the expression of Nrf2, and of its target genes such as HO-1 and SOD1, eliminating the neuroprotective effects of SIRT1 against a transient focal cerebral ischemia [87]. In addition, the modulation of SIRT1/Nrf2 signaling has been suggested to underlie the anti-aging and neuroprotective activity of various natural compounds [88,89,90,91,92]. For example, the grape antioxidant resveratrol, which is considered to be an activator of SIRT1 [93], has also been shown to modulate Nrf2-dependent antioxidant protein expression promoting neuroprotection against cerebral ischemic injuries [94]. 

After demonstrating the interaction among SIRT1, PGC1α, and Nrf2 and their modulation by 24-OHC, tau ubiquitination and total tau levels have been also shown (Figure 5A). It is likely that 24-OHC promotes tau ubiquitination triggering its deacetylation; indeed, we have previously shown in vitro that 24-OHC increases the deacetylation of tau, reducing the levels of phosphorylated tau through SIRT1 up-regulation [39]. The key role of Nrf2 in favoring tau ubiquitination and degradation was demonstrated using a specific Nrf2 siRNA (Figure 5B). Using the same Nrf2 siRNA, the involvement of Nrf2 in the induction of the proteasome activity was also demonstrated (Figure 6A). The possible degradation of the ubiquitinated tau via proteasome was confirmed by co-incubating the SK-N-BE cells with 24-OHC and MG-132, an inhibitor of proteasome activity: the proteasome inhibitor prevented the degradation of tau protein induced by 24-OHC (Figure 6B,C). 

Clearly, these data point out that the UPS is a critical proteolytic mechanism to prevent the accumulation in neurons of hyperphosphorylated tau that leads to NFT formation in the brain, as well as of aggregated Aβ proteins, avoiding neuron death; these data are in accordance with other studies [95,96,97]. Indeed, the inhibition of UPS activity is sufficient to induce neuron degeneration and death. In a study on short-post-mortem-interval autopsied AD brains, a significant decrease in proteasome activity was observed in the brain regions that showed more degenerative changes. The loss of proteasome activity was not associated with a decrease in proteasome expression, suggesting that the proteasome may become inhibited in AD by a post-translational modification [98]. Moreover, to examine the effects of tau ubiquitination on its solubility properties, human embryonic kidney 293 cells (HEK293) were co-transfected with tau or ubiquitin, followed by treatment with N-acetyl-Leu-Leu-Norleu-al (ALLN) or MG-132, both proteasome inhibitors. The polyubiquitinated tau localized to the insoluble fraction where its accumulation was enhanced by proteasomal inhibition using ALLN or MG-132, confirming that tau is degraded by the proteasome [99]. In another study, the treatment of primary neurons with HSP90 inhibitors to interrupt the proper chaperoning of tau, decreased tau levels. The addition of MG-132 completely blocked HSP90 inhibitor-mediated reduction in total tau and phosphorylated tau. This result suggests that, in the case of proteasome dysfunction, autophagy takes over to ensure tau homeostasis; however, if the proteasome damage overcomes the autophagic compensation, for example as a consequence of HSP90 involvement, autophagy is no longer capable of guaranteeing sufficient degradation of tau [100]. 

Despite this evidence, we cannot exclude the involvement of other mechanisms in tau homeostasis, such as autophagy. Indeed, while the UPS is responsible for the degradation of the full-length tau, the fragments of the protein seem to be cleared by the autophagy system [101,102]. Of note, in our experimental model the treatment with the UPS inhibitor MG-132 alone did not lead to tau accumulation, thus suggesting that other degradative machineries can intervene in the case of UPS failure. The accumulation of hyperphosphorylated tau and tau aggregates is associated to an impairment of both these degradative systems, contributing to neurodegeneration. Delineating how these pathways may be compromised in AD and how this contributes to tau pathology is of great importance and could have significance for developing new therapeutic approaches to prevent tau protein accumulation or to accelerate the clearance of tau [103]. In relation to this, it has been demonstrated that proteasome activity, but not its levels, decreased in specific AD-sensitive brain regions (i.e., hippocampus and parahippocampal gyrus, temporal gyri, and parietal lobule) compared to unaffected regions [98,104]. Additionally, tau appeared to be assembled with the proteasome in AD brain tissues; this suggests that tau, although being a target of the proteasome, cannot be completely degraded [104]. 

To prove the efficacy of reducing tau levels in the brain to counteract neurodegeneration, antisense oligonucleotides that selectively decrease tau mRNA and protein have been tested in mice carrying human tau. Lowering total tau levels were not only capable of preventing and reversing tau pathology, but cell survival was also significantly extended and neuronal loss abrogated [105]. High tau levels not only promote its own aggregation but can also mediate the toxicity of Aβ_42_ [19,20,22,106,107]. In relation to this, it has been demonstrated that tau reduction by gene knockout could hinder Aβ-induced deficits in axonal transport [108] and ameliorate the cognitive defects in mice models without influencing baseline neuronal functions [109]. Another promising strategy to induce non-enzymatic protein degradation inside the cell is the use of proteolysis targeting chimeras (PROTACs). Chu and colleagues designed and synthesized a multifunctional PROTAC, namely TH006, that was able to increase ubiquitination of tau and then its degradation; the partial reduction in tau induced by TH006 counteracted the Aβ_42_-induced cytotoxicity [110]. Recently, Jiang’s group developed and synthesized another peptide PROTAC (Peptide 1) by recruiting KEAP1-Cul3 ubiquitin E3 ligase and tau. This PROTAC could promote the KEAP1-dependent poly-ubiquitination and proteasome-dependent degradation of tau [111]. The results suggested that PROTACs may be considered promising therapeutic drugs in the treatment of tauopathies, including AD. 

With the aim to confirm the potential role played by the SIRT1/PGC1α/Nrf2 pathway in neuroprotection, the expression and the protein levels of the single molecules involved were investigated in the frontal and occipital cortices of AD patients at different stages of progression. We chose the frontal and occipital cortex for the study because these areas of the neocortex are affected by tau pathology in advanced AD stages (V–VI) but not in early ones (I–III). On the other hand, all the areas of the cerebral cortex show Aβ deposits already in the initial phases of the morphogenesis of the hallmark lesions, referred to as Braak stage I-III of neurofibrillary pathology. This choice allows us to compare a state where tau pathology is not yet present to ones where tau is markedly accumulated.

The gene expression and the protein levels of SIRT1 were markedly reduced with the progression of the disease in both frontal and occipital cortices, compared to the control brain specimens (Figure 7A and Figure 8A). Concerning PGC1α and Nrf2, the gene expression increased with the progression of AD (Figure 7B,C). Their over-expression may be a compensatory mechanism due to accumulation of oxidative stress-related species that, together with inflammation, contributes to the pathogenesis of AD. Indeed, the two transcription regulators are involved in ROS defense systems. However, an increase in the expression does not necessarily imply an increase in the protein levels. In this connection, our data have indicated a marked and progressive decrease in Nrf2 protein levels in cortices of AD patients with the progression of the disease (Figure 8C) while the protein levels of PGC1α were slightly decreased in the Early AD (Figure 8B). The reduction in the synthesis of the last two molecules could be the consequence of post-transcriptional modifications or positive feedback loops. We can thus hypothesize that the SIRT1/PGC1α/Nrf2 pathway might contribute to the neuroprotection by preventing the accumulation of tau in AD, a key feature of the disease. 

Other studies have also reported the reduction in SIRT1 and Nrf2 in AD brain, highlighting their potential neuroprotective action. Reduced SIRT1 expression levels were correlated with elevated Aβ production and deposition in AD patients [77] and loss of SIRT1 was closely associated with exacerbated Aβ production [112]. Moreover, the absence of SIRT1 expression in hippocampal neurons was correlated with impaired cognitive abilities, including immediate memory, classical conditioning, and spatial learning [113].

In addition, SIRT1 lower levels in the brain of AD patients are associated with the progression of the disease. In post-mortem human brain samples from AD patients, the levels of SIRT1 in some brain regions (entorhinal, temporal, and frontal cortices, hippocampus, and white matter) were lower than those in the corresponding control group, and significant correlations were observed between SIRT1 levels and Braak stages of neurofibrillary degeneration in the same regions of the brain [34,114,115]. In another study, the levels of SIRT1 were analyzed in brains obtained from subjects with a pre-mortem diagnosis of no-cognitive impairment (NCI), mild cognitive impairment (MCI), mild to moderate AD (mAD), and severe AD (sAD). SIRT1 decreased in MCI, mAD, and sAD compared to NCI and positively correlated with perceptual speed, episodic memory, global cognitive score, and Mini-Mental State Examination; moreover, SIRT1 negatively correlated with cortical neurofibrillary tangle counts [116]. The levels of SIRT1 were also analyzed in various brain regions of transgenic mice expressing the human apolipoprotein E4 (apoE4) isoform, which is consistently associated with increased risk for AD: SIRT1 levels were significantly reduced in the frontal cortex and its decrease may play a role in apoE4-associated memory impairments [117]. Regarding Nrf2, a significant decrease in nuclear Nrf2 levels in AD brains was shown by immunoblotting, suggesting that Nrf2-mediated gene transcription is deficient in neurons despite the presence of oxidative stress [118]. Of note, to demonstrate that the decrease in Nrf2 plays a role in the cognitive deficits associated with AD, a genetic approach to remove the Nrf2 gene from APP/PS1 mice, a widely used animal model of AD, was developed. The lack of Nrf2 significantly exacerbates cognitive deficits without altering motor function [119]. In the same experimental model, the intrahippocampal injection of a lentiviral vector expressing Nrf2 improved spatial learning [120]. 

## 5. Conclusions

**Figure 9:**
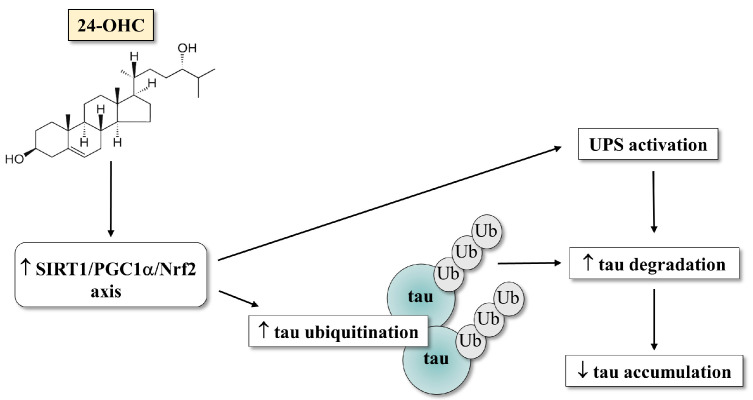
A hypothetical scheme for the proteasome degradation of tau by 24-OHC via SIRT1-dependent pathway.

In this study we demonstrated a beneficial role for 24-OHC in reducing tau accumulation in the SK-N-BE neuroblastoma cells by acting on the SIRT1/PGC1α/Nrf2 signaling pathway leading to UPS activation and consequently to tau degradation, as depicted in Figure 9. Of note, avoiding the intraneuronal accumulation of tau might prevent NFT formation and thus neuron death. Reduction in tau accumulation may thus open up a novel therapeutic strategy for AD treatment. Moreover, since the SIRT1/PGC1α/Nrf2 pathway appeared to be activated and sustained by 24-OHC, the reduction in the deacetylase SIRT1 expression in the AD brains with the disease worsening points out the importance of preventing the loss of 24-OHC in the brain, which was demonstrated to occur in AD progression [34], in order to maintain the activity of SIRT1-dependent pathways to counteract neurodegeneration.

## Figures and Tables

**Table 1:** Detailed information about the subjects included in the analyses.

Subject	Sex	Age (Years)	Post-Mortem (h)	Braak Stage	Thal Stage
Control (*n* = 9) 1 2 3 4 5 6 7 8 9	F F F F F M F M F	61 61 60 68 58 75 72 70 69	36 36 18 48 24 40 43 39 20	0 0 0 0 0 0 0 0 0	0 0 0 0 0 0 0 0 0
Early AD (*n* = 6) 10 11 12 13 14 15	M M F M F M	69 71 81 86 77 85	38 36 39 20 46 25	I I I II II III	1 1 1 1 1 3
Late AD (*n* = 8) 16 17 18 19 20 21 22 23	F F M M M F F F	67 82 75 79 77 58 72 81	28 21 26 45 16 48 34 37	V VI VI VI VI VI VI VI	4 4 4 4 4 4 4 4

Braak stage based on neurofibrillary changes; Thal stage based on Aβ deposits.

## Data Availability

The data presented in this study are available in the article or Supplementary Material.

## Supplementary Materials

The following supporting information can be downloaded at: https://www.mdpi.com/article/10.3390/antiox12030631/s1, Figure S1: Cytotoxicity of SR-18292 and MG-132 inhibitors. Figure S2: Validation of SIRT1 and Nrf2 gene silencing efficiency.
