# Supplementary material for: 24-Hydroxycholesterol Induces Tau Proteasome-Dependent Degradation via the SIRT1/PGC1α/Nrf2 Pathway: A Potential Mechanism to Counteract Alzheimer’s Disease
Source: Antioxidants (Basel). 2023 Mar 3;12(3):631. doi: 10.3390/antiox12030631 (PMC10044740; doi:10.3390/antiox12030631)
Supplement: Supplementary file 1 [file antioxidants-12-00631-s001.zip › Captions.pdf]

Figure S1. Cytotoxicity of SR-18292 and MG-132 inhibitors. SK-N-BE cells were treated for 24 h **(A)** with SR-18292, a PGC1α inhibitor, or **(B)** with MG-132, a proteasome inhibitor, at different concentrations. Cell viability was analyzed by MTT assay. Data represent mean values ± SD of 6 experiments and are expressed as percentage of control value (DMSO treated cells). * $p<0.05$, ** $p<0.01$, **** $p<0.0001$ vs. control.

Figure S2. Validation of SIRT1 and Nrf2 gene silencing efficiency. (**A**) SK-N-BE cells were transfected for 6 h with SIRT1 or scrambled siRNA. (**B**) SK-N-BE cells were transfected for 6 h with Nrf2 or scrambled siRNA. Transient SIRT1 or Nrf2 gene silencing was investigated by real-time RT-PCR. Data, normalized to the corresponding β_2-microglobulin levels, are expressed as mean values ± SD of 3 different experiments. ** $p<0.01$, **** $p<0.0001$ vs. control.
