# Supplementary figures and images for: 24-Hydroxycholesterol Induces Tau Proteasome-Dependent Degradation via the SIRT1/PGC1α/Nrf2 Pathway: A Potential Mechanism to Counteract Alzheimer’s Disease
Source: Antioxidants (Basel). 2023 Mar 3;12(3):631. doi: 10.3390/antiox12030631 (PMC10044740; doi:10.3390/antiox12030631)

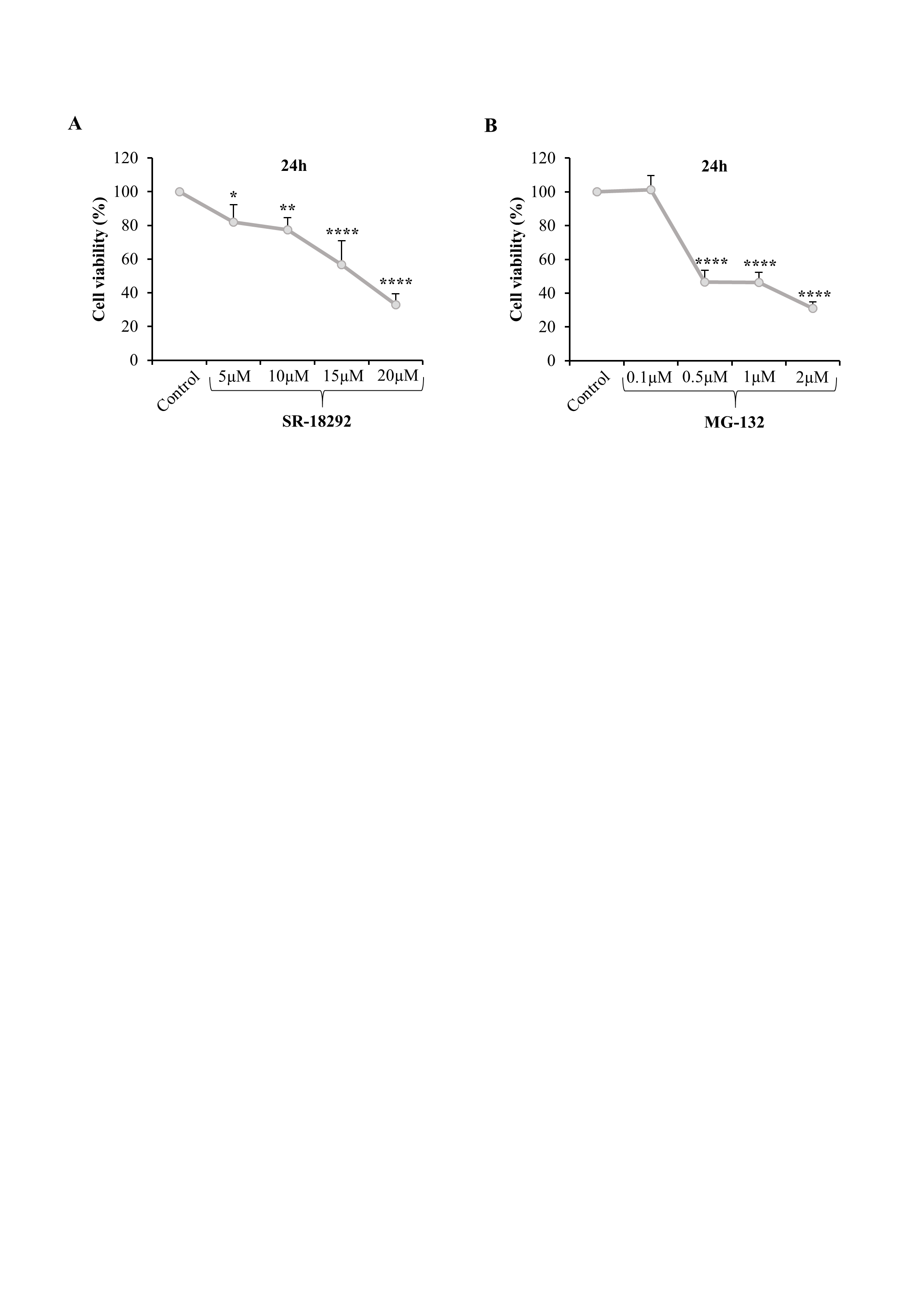

Supplement: Supplementary file 1 [file antioxidants-12-00631-s001.zip › Figure S1.tif]

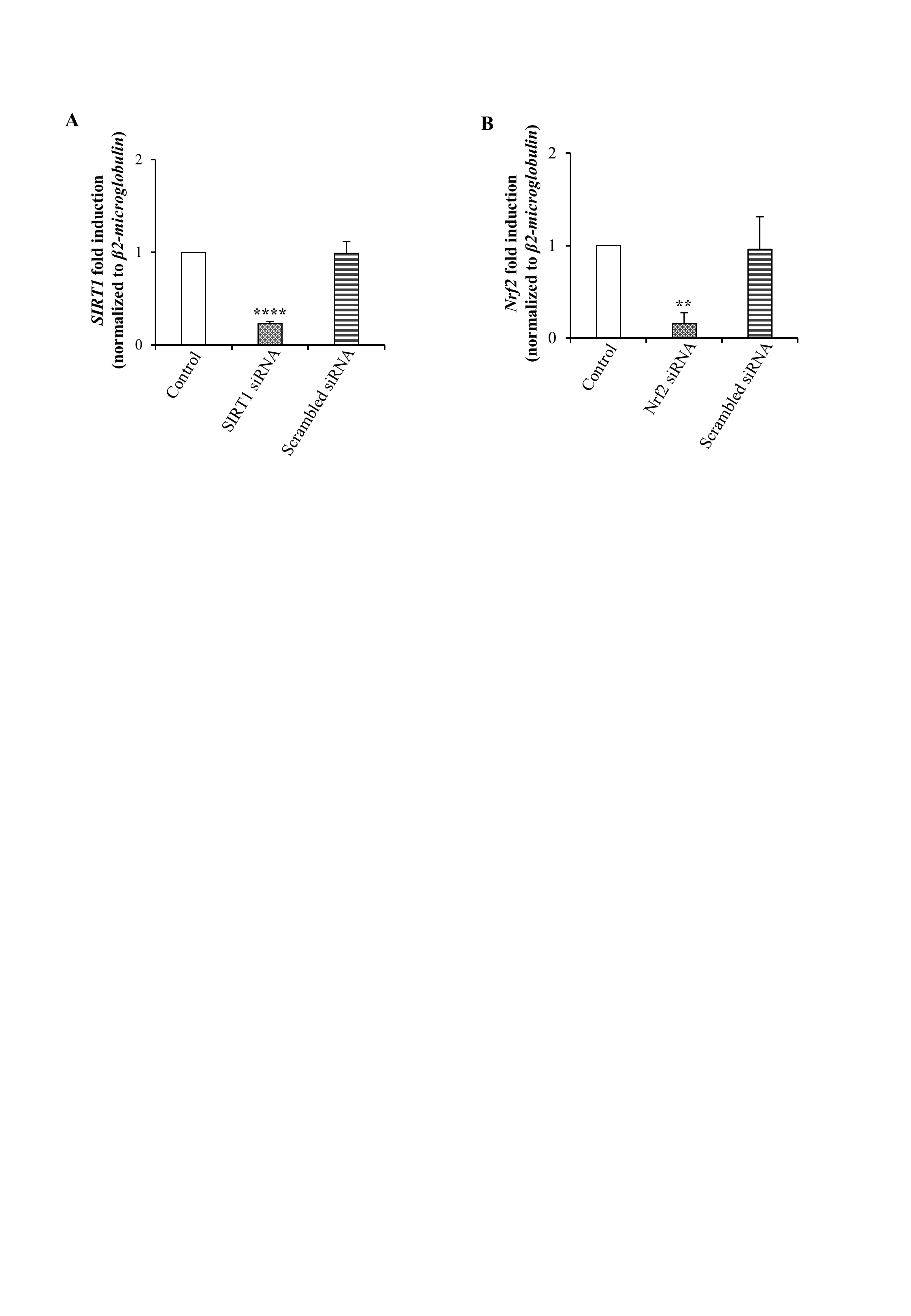

Supplement: Supplementary file 1 [file antioxidants-12-00631-s001.zip › Figure S2.tif]
